# A Combined NMR and UV–Vis Approach to Evaluate Radical Scavenging Activity of Rosmarinic Acid and Other Polyphenols

**DOI:** 10.3390/molecules28186629

**Published:** 2023-09-14

**Authors:** Arian Kola, Ginevra Vigni, Maria Camilla Baratto, Daniela Valensin

**Affiliations:** Department of Biotechnology, Chemistry and Pharmacy, University of Siena, Via Aldo Moro 2, 53100 Siena, Italy; arian.kola@unisi.it (A.K.); ginevra.vigni2@unisi.it (G.V.); mariacamilla.baratto@unisi.it (M.C.B.)

**Keywords:** rosmarinic acid, hydroxycinnamic acids, copper, NMR, antioxidant activity, prooxidant activity

## Abstract

Oxidative stress results from an imbalance between reactive oxygen species (ROS) production and the body’s ability to neutralize them. ROS are reactive molecules generated during cellular metabolism and play a crucial role in normal physiological processes. However, excessive ROS production can lead to oxidative damage, contributing to various diseases and aging. This study is focused on rosmarinic acid (RA), a hydroxycinnamic acid (HCA) derivative well known for its antioxidant activity. In addition, RA has also demonstrated prooxidant behavior under specific conditions involving high concentrations of transition metal ions such as iron and copper, high pH, and the presence of oxygen. In this study, we aim to clarify the underlying mechanisms and factors governing the antioxidant and prooxidant activities of RA, and to compare them with other HCA derivatives. UV–Vis, NMR, and EPR techniques were used to explore copper(II)’s binding ability of RA, caffeic acid, and p-coumaric acid. At the same time, UV–Vis and NMR methods were exploited to evaluate the polyphenols’ free radical scavenging abilities towards ROS generated by the ascorbic acid–copper(II) system. All the data indicate that RA is the most effective polyphenol both in copper binding abilities and ROS protection.

## 1. Introduction

Oxidative stress is a complex biological phenomenon resulting from the imbalance between reactive oxygen species (ROS) production and the body’s ability to neutralize and eliminate them. ROS are highly reactive molecules containing oxygen that are naturally generated during cellular metabolism. Under normal circumstances, the body’s antioxidant defense systems effectively counteract ROS, maintaining cellular redox balance [1]. ROS are chemically unstable molecules including superoxide anion (•O_2_^−^), hydrogen peroxide (H_2_O_2_), hydroxyl radical (•OH), and singlet oxygen (^1^O_2_). They are produced mainly as byproducts of cellular respiration in the mitochondria, as well as through enzymatic reactions in various cellular compartments such as peroxisomes and the endoplasmic reticulum. The body possesses a complex network of antioxidant defense mechanisms to neutralize ROS and prevent oxidative damage, which include enzymatic (e.g., superoxide dismutase, catalase, glutathione peroxidase) and non-enzymatic molecules [2,3,4,5,6,7] (e.g., vitamin C, vitamin E, glutathione). Together, they work synergistically to scavenge ROS and maintain cellular redox homeostasis [8,9].

Oxidative stress can inflict damage on lipids, proteins, and nucleic acids, leading to alterations in cellular structure and function. Oxidative damage to DNA can result in mutations and contribute to the development of cancer [10]. Protein oxidation may disrupt enzymatic activities and participate in protein misfolding, impacting cellular functions [11,12]. Lipid peroxidation can compromise cell membrane integrity, leading to cell dysfunction and death. The cumulative effects of oxidative stress have significant implications for human aging processes [13]. Chronic oxidative stress is associated with the pathogenesis of several diseases, including Alzheimer’s disease [14], Parkinson’s disease [14,15,16], cardiovascular diseases [2,17], and diabetes [18].

Polyphenols, a class of bioactive compounds widely distributed in plant-based foods and beverages, have attracted substantial scientific interest due to their recognized antioxidant properties [19,20] and potential health benefits [21,22,23,24].

**Figure 1:**
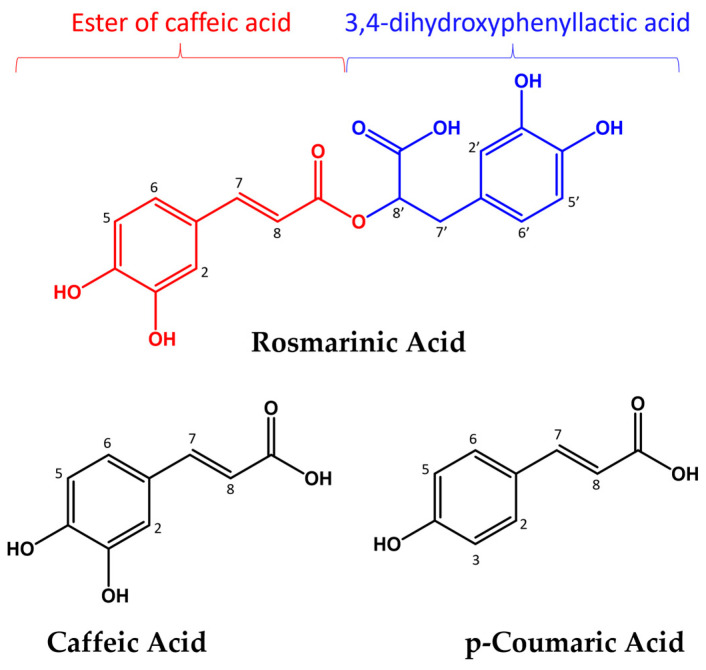
Chemical structures of RA, CA, and pCA polyphenols.

Among them, rosmarinic acid (RA) is a phytocompound abundantly found in various botanical sources such as rosemary (Rosmarinus officinalis), sage (Salvia officinalis), and lemon balm (Melissa officinalis). RA belongs to the group of hydroxycinnamic acids (HCA), and its chemical structure consists of two moieties: the ester of caffeic acid and 3,4-dihydroxyphenyllactic acid (Figure 1). RA has shown remarkable antioxidant properties in vitro and in vivo [25,26,27], and its effectiveness in neutralizing free radicals has been widely documented [28,29,30,31]. Its important ability to capture free radicals is attributed to the presence of hydroxyl groups in the phenolic rings, which allows it to donate and accept electrons, thus stabilizing radical species and preventing cell damage. Transition metal ions, particularly iron and copper, can participate in the Fenton and Haber–Weiss reactions, leading to the generation of highly reactive hydroxyl radicals [32,33]. RA can chelate these metal ions, sequestering them and preventing their participation in the generation of harmful ROS.

However, recent investigations have shed light on an intriguing and paradoxical aspect of RA’s behavior, that is, its capacity to act as a prooxidant under specific conditions, including high concentrations of transition metal ions, alkali pH, and the presence of oxygen molecules [34,35]. In particular, redox active metal ions such as Fe(III) and Cu(II) are capable of catalyzing oxidative reactions alone or in the presence of other compounds. Many scientific studies have shown the correlation between the pathological state and the increased concentration of these transition metals. For instance, cancer cells have a higher exigency of copper ions compared to the healthy cells [36,37,38,39]. In addition, the high serum level of copper is related to an increasing risk of atherosclerotic disease. Moreover, the human atherosclerotic plaques are characterized by an elevated content of copper [40], whereas most neurodegenerative diseases share dysregulated cellular copper homeostasis [40].

The antioxidant activity of RA and other HCAs has been observed in various experimental settings, but the underlying mechanisms and factors governing this behavior remain unclear. In our research, UV–Vis, NMR, and EPR techniques were exploited to obtain more insight into copper’s interaction with RA and two other HCAs: caffeic acid (CA) and p-coumaric acid (pCA) (Figure 1). Metal titration experiments were conducted to determine the polyphenols’ ligand ability and to elucidate the role played by the hydroxyl groups in Cu(II) coordination. Additionally, their ability to scavenge free radicals was investigated using UV–Vis and NMR spectroscopies. To explore the antioxidant properties, we employed ascorbic acid (AH) and Cu(II) ions able to generate ROS upon HA oxidation (vide infra) [41,42,43]. The choice of this system was mainly due to the following: (i) redox active metals ions, such as copper, are known to be involved in oxidative stress, and (ii) they promote the prooxidant activity of AH, which is an abundant antioxidant molecule present in human body [44]. Finally, NMR investigations provided an extensive characterization of all the species formed in solution upon AH oxidation, offering valuable insights into the antioxidant behavior of the three polyphenols.

## 2. Results

### 2.1. Copper(II) Interaction with Rosmarinic Acid (RA), Caffeic Acid (CA) and p-Coumaric Acid (pCA)

**Figure 2:**
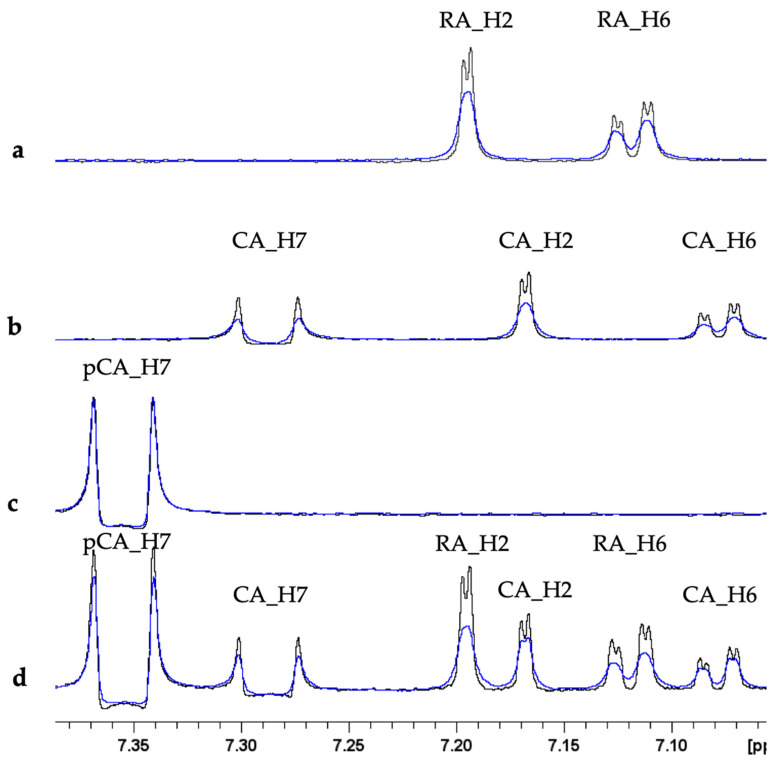
Superimposition of selected regions of ^1^H NMR spectra of (**a**) RA; (**b**) CA; (**c**) pCA; and (**d**) RA + CA + pCA in absence (black) and in presence of 0.16 (blue) Cu(II) eqs. In each sample, all the polyphenols have a concentration of 0.5 mM, while the phosphate buffer is 20 mM, pH 7.5, T = 298 K.

The interaction between polyphenols and Cu(II) has been extensively explored in the last years [45,46]. Copper binding occurs via OH groups generally leading to the formation of bis-complexes [45]. In order to correlate and compare copper binding abilities among different polyphenols, the behavior of RA, CA, and pCA was investigated by using NMR, EPR, and UV–Vis spectroscopies. NMR analysis was performed by adding sub-stoichiometric Cu(II) amounts to polyphenol solutions. As expected, the presence of copper caused selective line broadening on proton resonances because of the electron–nucleus dipolar coupling [47,48,49,50,51,52]. The largest effects were detected on RA and CA only, while almost no variations were shown by pCA (Figure 2). The copper-induced effects on HCAs protons were also monitored by measuring the intensity ratio I/I_0_ reported in Table 1, where I_0_ and I are the intensities of the NMR signal in the absence and in the presence of 0.16 Cu(II) eqs., respectively. I/I_0_ values close to 1, as detected for pCA, indicate no changes on NMR signal intensity, while values smaller than 1, observed for RA and CA, indicate intensity decrease induced by the paramagnetic ion. Furthermore, metal titration experiments performed on a solution containing equimolar amount of the three polyphenols made it possible to identify the strongest ligand among RA, CA, and pCA. As shown in Figure 2 and Table 1, RA maintains almost the same line-broadening and I/I_0_ values observed alone. On the other hand, the effects recorded on CA were less pronounced in the polyphenol mixture, thus suggesting that copper is preferentially coordinated to RA.

**Figure 3:**
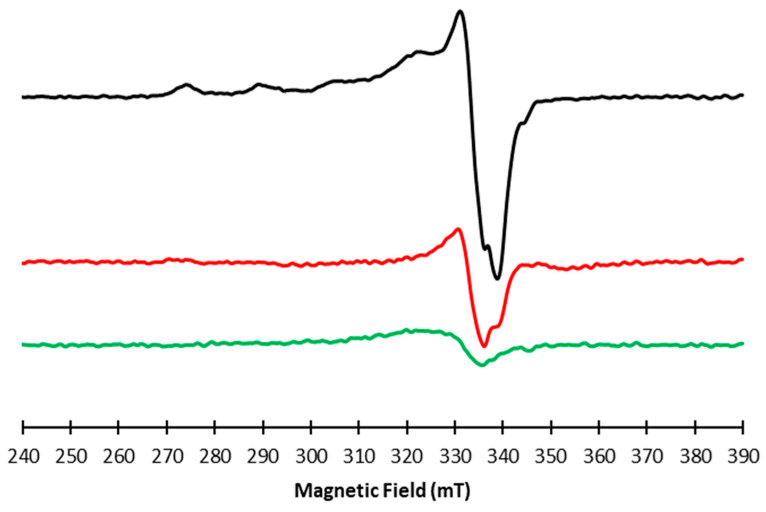
140 K X-band spectra of RA–Cu(II) (black trace), CA–Cu(II) (red trace), and pCA–Cu(II) (green trace) complexes recorded at ν = 9.67 GHz, microwave frequency, 0.5 mT modulation amplitude, 21 mW microwave power.

From the analysis of the EPR spectra recorded at low temperature, 140 K, and reported in Figure 3, it is evident that a Cu(II) complex is formed and detectable for RA and CA. For the pCA species, instead, the complex formation is very low, as reported by the green line: even if the intensity is low, the signal overlaps with the one of the complexes formed for RA and CA. In order to characterize the species formed, we focused on the low temperature spectrum of RA, as it is the best resolved and the most intense spectrum. The line shape of the EPR spectrum is typical of an axial geometry: the parallel region is visible, and the magnetic parameters calculated and then refined by the best fitting simulations are: g_‖_ = 2.324, g⊥ = 2.069, and A_‖_ = 15.1 mT. The simulation was performed considering no nitrogen atom in the coordination sphere of Cu(II) and this is in agreement with the Peisach and Blumberg diagram reported for four oxygen atoms coordinated to the Cu(II) center [53]. The experimental spectrum of the RA complex paired to its best fit simulation is reported in Figure S1 (Supplementary Materials). The EPR parameters obtained for the RA–Cu(II) complex are in line with previous published ones [28], confirming the formation of the bis copper complex. From an accurate analysis of the parallel region of the spectrum, the contemporary presence of more than one species cannot be excluded, due to the very low intensity appearance of additional peaks in the parallel region. Unfortunately, the magnetic parameters of such contribution cannot be calculated because they are not well resolved, preventing the possibility of simulation of a second complex.

The UV–Vis spectra were measured for the CA–Cu(II) system only since UV–Vis data for the RA–Cu(II) complex were previously collected by our group [28]. On the other hand, the lack of any relevant copper–pCA binding, monitored by both NMR and EPR spectroscopies, led us to exclude pCA from further studies. As for RA, metal titration UV–Vis experiments were performed (Figure S2). Copper additions caused no significant variations on the free ligand spectrum, contrary to RA, where metal chelation ended up with the appearance of new electronic absorptions [28].

### 2.2. Evaluation of the ROS Scavenging Abilities of Rosmarinic Acid (RA), Caffeic Acid (CA), and p-Coumaric Acid (pCA)

#### 2.2.1. UV–Vis Monitoring of Ascorbate Oxidation Promoted by Copper(II)

**Figure 4:**
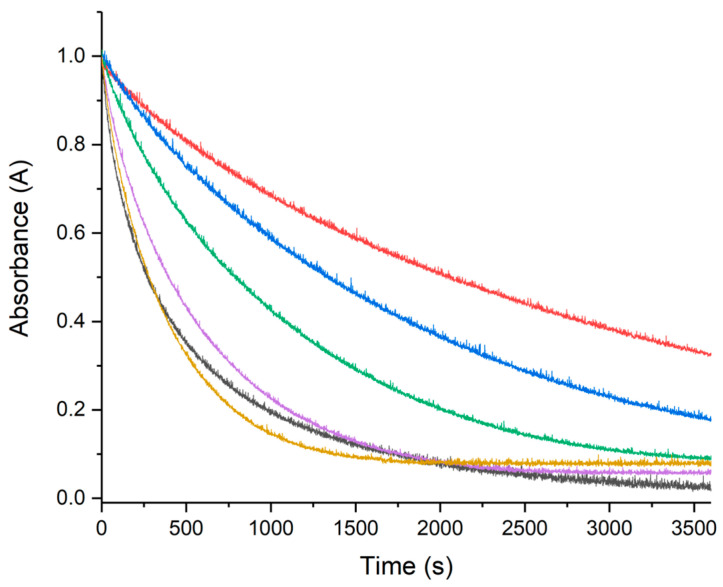
UV–Vis kinetic curves of the systems composed of AH 50 μM, Cu(II) 0.5 μM, and RA at different concentrations. In the gray curve, we can see the behavior of ascorbate in the presence of only copper(II), while the other colors correspond to the trend recorded in the presence of increasing RA concentrations. Specifically, RA 0.5 μM (yellow), RA 1 μM (violet), RA 5 μM (green), RA 10 μM (blue), and RA 25 μM (red).

**Figure 5:**
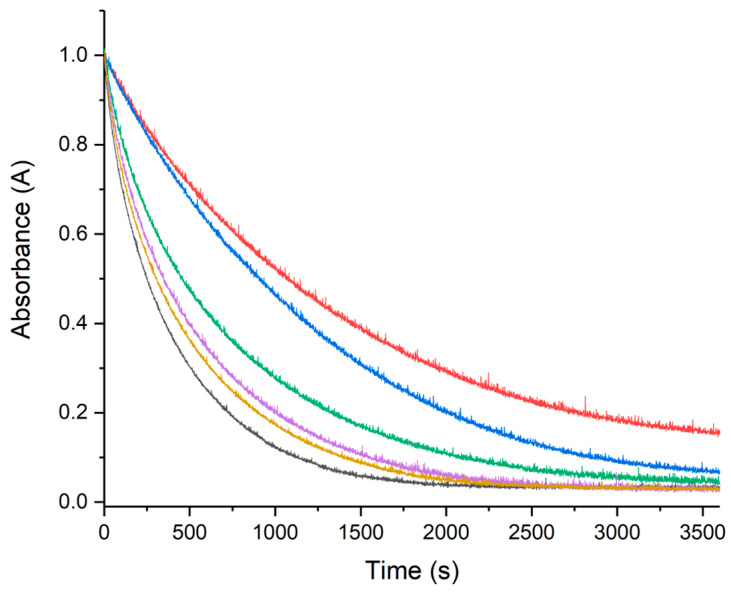
UV–Vis kinetic curves of the systems composed of AH 50 μM, Cu(II) 0.5 μM, and CA at different concentrations. In the gray curve, we can see the behavior of ascorbate in the presence of only copper(II), while the other colors correspond to the trend recorded in the presence of increasing CA concentrations. Specifically, CA 0.5 μM (yellow), CA 1 μM (violet), CA 5 μM (green), CA 10 μM (blue), and CA 25 μM (red).

**Figure 6:**
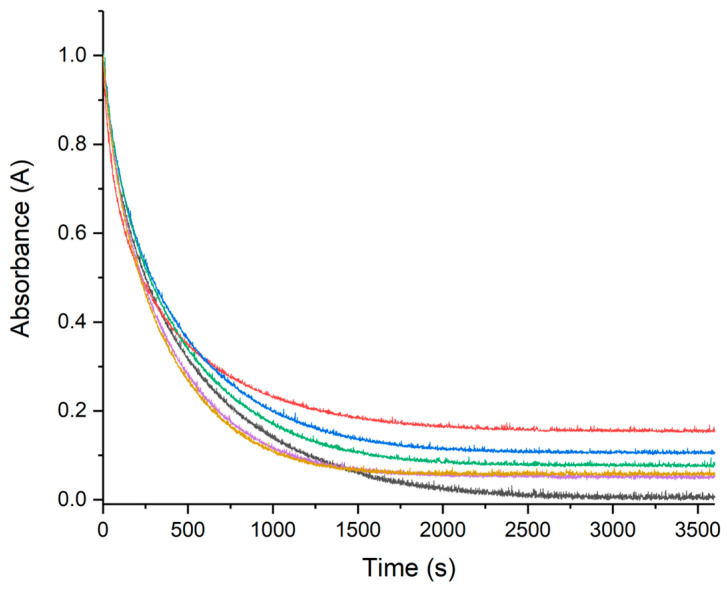
UV–Vis kinetic curves of the systems composed of AH 50 μM, Cu(II) 0.5 μM, and pCA at different concentrations. In the gray curve, we can see the behavior of ascorbate in the presence of only copper(II), while the other colors correspond to the trend recorded in the presence of increasing pCA concentrations. Specifically, pCA 0.5 μM (yellow), pCA 1 μM (violet), pCA 5 μM (green), pCA 10 μM (blue), and pCA 25 μM (red).

Metal ions such as Cu(II) and Fe(III) are able to accelerate the oxidation of ascorbate (AH) in presence of oxygen, leading to the production of ROS through Fenton-type reactions [54,55,56,57]. The ascorbate consumption is usually monitored by looking at its absorption at 265 nm as a function of time, giving a typical kinetic curve with a slope directly dependent on the reaction rate. The faster the AH oxidation (consumption), the higher the ROS production. From this perspective, the influence of RA, CA, and pCA on HA oxidation were measured to evaluate the role played by these three polyphenols as ROS scavenger. The kinetic curves collected for RA, CA, and pCA at different concentrations are shown in Figure 4, Figure 5 and Figure 6, respectively. Increasing polyphenol concentration yields a slower AH consumption in all the cases. The AH oxidation is delayed with the following order RA > CA > pCA, suggesting that among the three compounds, RA has the strongest protective activity. In order to avoid any possible contributions coming from UV–Vis polyphenol absorptions, the electronic behavior of all three polyphenols in the presence of Cu(II) was monitored as well. The spectra recorded for fifteen minutes were completely unchanged, thus confirming their stability.

#### 2.2.2. NMR Monitoring of Ascorbate Oxidation Promoted by Copper(II)

**Figure 7:**
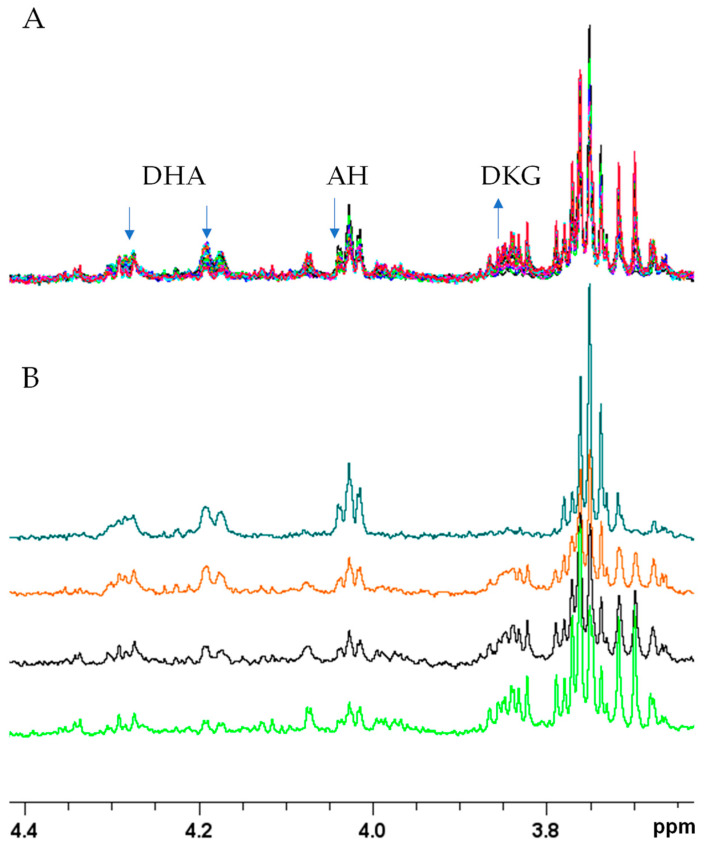
^1^H NMR spectra of AH 0.5 mM, Cu(II) 5 × 10^−3^ mM, and phosphate buffer 20 mM at different time intervals. (**A**). Superimposition of all thirty spectra collected after 1 h. (**B**). Superimposition of spectra at t = 0 min (dark green), t = 20 min (orange), t = 40 min (black), and t = 60 min (light green).

The AH–Cu(II) reactivity was also investigated through NMR spectroscopy, which is able to detect both the consumption and the production of AH-related compounds (Figure 7). The first recorded NMR spectrum corresponds to the sample after 5 min (mins) from the Cu(II) addition and it was followed by thirty NMR spectra lasting 2 min each, for a total duration of 60 min. The initial 5 min were necessary for setting the homogeneity of the magnetic field, tuning the proton frequency, and defining the experimental conditions. The same procedure was also applied to all the other samples described in the following paragraphs. For the sake of clarity, in the next sections and figures, we will refer to the first NMR spectrum recorded after 5 min as time = 0.

Figure 7 shows the presence of the signals of both AH and its oxidized form, dehydroascorbate (DHA), in the spectra at t = 0 min. Over time, both signals decreased to almost disappear after 1 h. Interestingly, at about 10 min, the presence of new NMR resonances was detected, and their intensity increased until the end of the kinetic. The chemical shifts of those signals are consistent with the data reported in human metabolome database for the predicted 900 MHz NMR spectrum of diketogulonic acid (DKG) [58]. The occurrence of DKG in solution is also in agreement with the fact that DHA can undergo hydrolysis to produce 2,3-diketogulonic acid [55,59,60,61].

**Figure 8:**
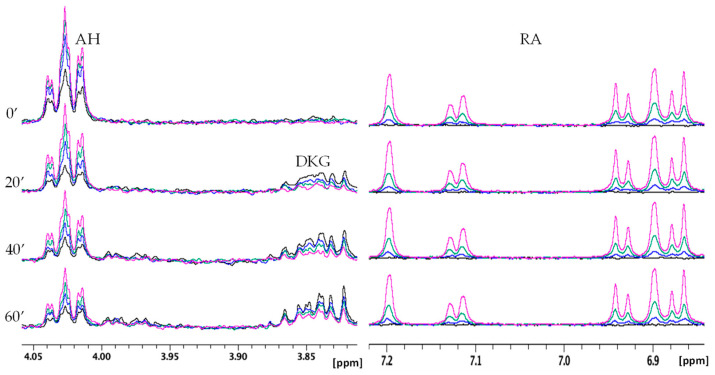
Selected regions of time-dependent ^1^H NMR spectra of AH 0.5 mM, Cu(II) 5 × 10^−3^ mM, phosphate buffer 20 mM in absence (black) and in presence of RA 0.05 mM (blue), 0.125 mM (green), and 0.25 mM (magenta). AH and RA signals are shown on the left and right, respectively.

**Figure 9:**
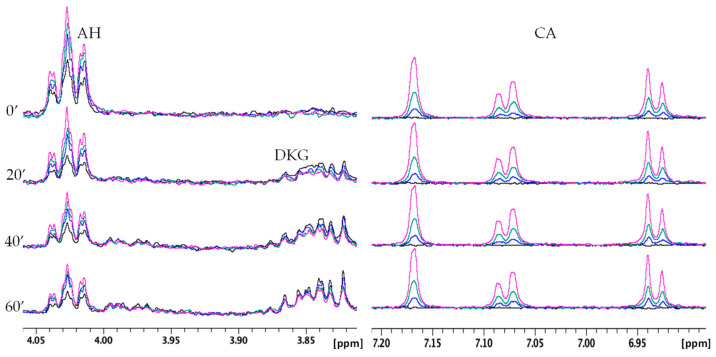
Selected regions of time-dependent ^1^H NMR spectra of AH 0.5 mM, Cu(II) 5 × 10^−3^ mM, phosphate buffer 20 mM in absence (black) and in presence of CA 0.05 mM (blue), 0.125 mM (green), and 0.25 mM (magenta). AH and CA signals are shown on the left and right, respectively.

**Figure 10:**
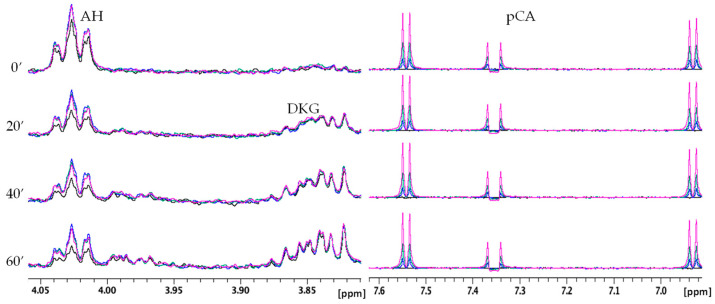
Selected regions of time-dependent ^1^H NMR spectra of AH 0.5 mM, Cu(II) 5 × 10^−3^ mM, phosphate buffer 20 mM in absence (black) and in presence of pCA 0.05 mM (blue), 0.125 mM (green), and 0.25 mM (magenta). AH and pCA signals are shown on the left and right, respectively.

In order to evaluate the influence of RA, CA, and pCA on the AH oxidation products, NMR spectra were also recorded in the presence of different polyphenol concentrations (Figure 8, Figure 9 and Figure 10). For all three cases, the amount of RA, CA, and pCA was set to 0.5, 0.25, and 0.1 equivalents with respect to AH concentration. Figure 8 shows the data obtained for RA, where the higher the polyphenol concentration, the lower the AH consumption and DKG formation. In addition, the invariance detected on the aromatic signals of RA indicates the polyphenol’s stability over all the kinetic time. The NMR signals of RA were also compared with the ones recorded in the same conditions but in the absence of AH. At all the tested concentrations, the resonances of RA are well superimposed (Figure S3), strongly supporting the lack of AH interference in the interaction between RA and Cu(II).

Very similar NMR behavior was observed for CA, since, like RA, it is able to modulate AH oxidation reactions (Figure 9). On the other hand, pCA exhibited less evident effects than the other two polyphenols in agreement with the UV–Vis data (Figure 10).

## 3. Discussion

As previously mentioned, vitamin C, also known as ascorbic acid (AH), is one of the most important radical scavenging systems utilized by the human body. However, under certain circumstances, whether in the presence or absence of iron and copper, it can also strongly act as a prooxidant molecule [37,38,62,63,64,65]. The antioxidant activity of vitamin C is explained by its self-oxidation, forming intermediate radicals with low activity. In this way, it can combine with highly reactive radicals to form less reactive ones, such as the ascorbate radical. Although this process may be influenced by factors such as concentration and pH [66,67,68], the ascorbate radical still remains thermodynamically close to the bottom of the pecking order for oxidizing radicals, as proposed by Buettner and co-workers in 1993 [69]. Consequently, it tends to neutralize some more reactive species, including •OH (hydroxyl radical) and •O_2_^−^ (superoxide anion) [62]. On the other hand, the prooxidant property of AH can emerge in the presence of iron or copper ions, leading to the generation of ROS [70]. The mechanism of the reaction is still unclear, but it is generally accepted to define it as a catalytic rather than a redox one [55]. In fact, even with small traces of copper, AH can reduce O_2_ to H_2_O_2_ with the following mechanism (Equation (1)) [50]:

AH_2_/AH^−^ + Cu(II) + O_2_ → DHA + Cu(II) + H_2_O_2_ (−H^+^) (1)

Copper ions and H_2_O_2_ can participate in a Fenton reaction, where Cu(I) is re-oxidized to Cu(II), and hydrogen peroxide is utilized to produce the hydroxyl radical. Overall, AH generates H_2_O_2_ and •OH from O_2_ [71,72]. In addition to the generation of ROS, the oxidation of AH also produces other molecules, including dehydroascorbic acid (DHA), 2,3-diketogulonic acid (DKG), and other oxidative compounds [59].

In this study, we investigated the ability of RA to scavenge ROS in the presence of Cu(II) ions and a reducing agent, ascorbic acid. The behavior of RA was then compared to that of CA and pCA, all belonging to the class of hydroxycinnamic acids. In addition to the UV–Vis technique, commonly used in this type of study, we utilized NMR spectroscopy, which has not been used before, to the best of our knowledge, to evaluate the radical scavenging activity of natural polyphenols.

The combined employment of these two methods allowed us to monitor multiple aspects of the reaction, including (i) the consumption of ascorbic acid over time, (ii) the formation of oxidation products such as DHA and DKG, and (iii) the stability of polyphenols over time and their potential impact on the AH–Cu(II) system. Furthermore, the application of NMR spectroscopy can be highly useful in investigating systems involving molecules with UV–Vis absorption frequencies close to the AH one (265 nm), which is typically monitored in UV–Vis spectroscopy.

The data from our study indicate that all three investigated polyphenols are capable of interfering with the ascorbate–Cu(II) system, leading to a decrease in AH–DHA oxidation. However, pCA exhibited a much lesser influence on AH oxidation compared to the other two HCAs. In fact, the results obtained from both UV–Vis and NMR spectroscopy agree in highlighting the following order of effects: RA > CA > pCA. At the same time, NMR and EPR analyses indicate that only RA and CA can effectively coordinate the cupric ion (Cu(II)) through the hydroxyl groups of their polyphenolic moieties. The coordination bond becomes more efficient as the number of available OH groups increases. Once again, RA proves to be the most effective among the three investigated polyphenols in establishing this coordination bond with the cupric ion.

In light of these results, the slowdown in AH consumption observed in the presence of RA and CA may be attributed to the ability of both polyphenols to coordinate Cu(II) ions, thereby sequestering it and preventing its participation in the reaction with AH. Simultaneously, it is also possible to hypothesize the formation of a ternary complex, involving AH, Cu(II), RA, or CA, which leads to significant changes in the kinetics of the Cu(II) –AH reaction. In a similar manner, the formation of a ternary complex has been proposed for the systems AH–Cu(II)–Histidine, AH–Cu(II)–Amyloid-ß, and AH–Fe(III)–deferiprone [73,74].

On the other hand, the near absence of Cu(II)–pCA interaction allows us to directly evaluate the radical scavenging effect of the phenolic moiety. As depicted in Figure 6, the presence of pCA does not cause any variation in the curve during the initial kinetic phase (60–100 s), normally used to determine the kinetic rate [54,73]. The curve remains practically unchanged in both the absence and presence of different concentrations of pCA, indicating that the initial reaction rate of the AH–Cu(II) system is not affected by pCA. Figure 6 highlights that pCA’s influence is predominantly observed in the later stages of the reaction, suggesting that the polyphenol radical scavenging ability becomes more apparent as the reaction progresses. Indeed, distinct behaviors are observed in the later stages of the kinetics, depending on the concentration of pCA used. When pCA is present at concentrations of 0.25, 0.10, and 0.05 mM, it leads to a reduced AH consumption after approximately 5 min. However, lower pCA concentrations (0.01 and 0.005 mM) initially result in a decrease of AH levels between 5 to 15 min, followed by a slowdown in the reaction rate until the end of the kinetic time (Figure 6). A similar trend is observed in the NMR spectra (Figure 10), where the intensity of the ascorbate signal is higher at all the investigated pCA concentrations (0.05 mM, 0.125 mM, and 0.25 mM). In contrast, no changes are observed in the signals corresponding to DKG.

The distinct behaviors exhibited by RA, CA, and pCA strongly indicate the significance of polyphenol copper binding ability in exerting protective effects on ROS production. This behavior is mainly related to the fact that redox active metal ions, such as copper and iron, are able to catalyze the generation of ROS from molecular oxygen. The ability of RA to coordinate with copper ions plays a crucial role in its radical scavenging and antioxidant activities, ultimately leading to a reduction in ROS production and oxidative stress. This behavior suggests that Cu(II)–RA interactions are correlated with RA ability in combating oxidative damage and promoting cellular health, similarly to other polyphenols [75,76,77,78,79,80]. It is also interesting to emphasize that all the experiments were conducted using sub-stoichiometric concentrations of the copper ion, resembling the trace copper amount found in physiological conditions.

Finally, the antioxidant function demonstrated by RA in this study is further supported by the fact that RA has a protective role against cellular aging, apoptosis, and endothelial dysfunction induced by H_2_O_2_ [81,82,83,84,85,86], which is one of the major ROS produced by the AH–Cu(II) system.

## 4. Materials and Methods

### 4.1. Materials

CuSO_4_ solution (4% *w*/*v*, prepared from copper(II) sulfate pentahydrate), ascorbic acid (≥99%), caffeic acid (≥98.0% HPLC), rosmarinic acid (≥98.0% HPLC), and p-coumaric acid (≥98.0% HPLC) were all supplied by Sigma-Aldrich (Schnelldorf, Germany), which provided us the phosphate buffer too.

### 4.2. Sample Preparation

Ascorbate was diluted with distilled water to reach the final concentration in the stock solution of 10 mM. The polyphenols, instead, were solubilized in phosphate buffer 30 mM and then diluted with distilled water, to reach the stock final concentration of 0.5 mM and 5 mM for the UV–Vis and the NMR experimental parts, respectively. Concerning the Cu(II), the stoichiometric ratio between ascorbic acid and Cu(II) was set at 100:1; thus, a stock solution of CuSO_4_ in distilled water was prepared at the final concentration of 0.25 mM.

### 4.3. UV–Vis Measurements

All the stock solutions freshly prepared were diluted with phosphate buffer and distilled water in order to have, in a cuvette with a total volume of 500 μL, a final concentration of 50 μM for the ascorbic acid and 1 mM for the phosphate buffer. For the UV–Vis experimental part, the Cu(II) concentration was set at 0.5 μM; hence, polyphenols were introduced in the system too, whose quantity, in the final volume, changed in each experiment. Hereafter, the chosen hydroxycinnamic acid concentrations are reported: 25 μM, 10 μM, 5 μM, 1 μM, and 0.5 μM. The absorption spectra and the kinetic curves (60 min, 3600 s) were recorded on a Perkin Elmer Lambda 900 UV/VIS/NIR spectrophotometer.

### 4.4. NMR Experiments

NMR spectra and kinetics of 1 h were performed with a Bruker Avance III Spectrometer, at 14.1 T, and using a 5 mm BBI probe. All the experiments were collected and carried out at the controlled temperature of 298 K ± 0.2 K. Chemical shifts were referenced to external 2-(Trimethylsilyl)-propionic-2,2,3,3-d_4_ acid sodium salt (TMSP-d_4_). 1D spectra were recorded by using standard pulse sequences, and were analyzed by using the TopSpin 4.1.4 software. Residual water signal was suppressed by excitation sculpting pulse program, applying a selective 2 ms long square pulse on water [87]. Stock solutions of CA, RA, pCA (5 mM), AH (10 mM), and CuSO_4_ (0.25 mM) in phosphate buffer 20 mM pH 7.5 were freshly prepared and used in each experiment in a different quantity to reach the final desired stoichiometric ratio. For all the NMR analyses, the AH:Cu(II) ratio was set at 100:1, while the AH:polyphenol ratio was modified in each kinetic (10:1, 4:1, 2:1, and 1:1). Samples were all prepared in phosphate buffer 20 mM pH 7.5 with 10% D_2_O; from the stock solution, AH was diluted every time too in order to reach the concentration of 0.5 mM in the NMR tube.

### 4.5. EPR Spectroscopy

EPR measurements (CW X-band, 9 GHz) were carried out with a Bruker Elexsys Series E580 spectrometer using a Bruker ER4122 SHQE cavity, while the temperature was controlled by the Bruker ER4111t variable temperature unit. Simulations were run using the software for fitting EPR frozen solution spectra, which is a modified version of the program written by J.R. Pilbrow [88]. All the samples were prepared in a metal:ligand molar ratio 1:2 in buffer solution. For the low temperature measurements, glycerol was added to obtain a good glass during the freezing process.

## 5. Conclusions

In this study, we evaluated the antioxidant/prooxidant activity of rosmarinic acid, a polyphenol known for its effectiveness in countering oxidative stress-induced damage. The obtained results were then compared with those of two polyphenols belonging to the same class, hydroxycinnamic acids, i.e., caffeic acid and p-coumaric acid. The three polyphenols were also analyzed for their ability to bind copper ions. Our findings indicate that all three polyphenols act as antioxidants under the conditions used and that rosmarinic acid is the most effective one. The data obtained in the study indicate that rosmarinic acid has a greater ability to coordinate with copper ions compared to other polyphenols such as caffeic acid and p-coumaric acid. This higher affinity for copper may give rosmarinic acid a superior capacity to counteract the oxidation of ascorbic acid (vitamin C) in the presence of copper, thus protecting the body from excessive production of free radicals and oxidative stress. However, it is important to note that the effects of rosmarinic acid on copper coordination and its antioxidant activity may vary depending on the experimental conditions and the context within the body. Further studies may be necessary to delve deeper into this interaction and its impact on human health.

## Figures and Tables

**Table 1:** Chemical shift and copper induced intensity variations of proton NMR signals of RA, CA, and pCA alone and in the mixture.

Polyphenol	Proton	ppm	I/I_0_ Alone	I/I_0_ Mix
RA	H2	7.19	0.70	0.63
	H6	7.12	0.83	0.77
	H7	7.59	0.68	0.60
	H8	6.39	0.79	0.70
	H2′	6.90	0.86	0.77
	H5′	6.87	0.67	0.55
	H6′	6.80	0.63	0.49
CA	H2	7.17	0.56	0.86
	H6	7.08	0.61	0.89
	H7	7.29	0.52	0.80
	H8	6.35	0.55	0.75
pCA	H2	7.54	1.01	0.95
	H6	7.54	1.01	0.95
	H7	7.35	1.01	0.98
	H8	6.38	1.00	0.98

## Data Availability

The data presented in this study are available in this article and the Supplementary Materials.

## Supplementary Materials

The following supporting information can be downloaded at: https://www.mdpi.com/article/10.3390/molecules28186629/s1, Figure S1: 140 K X-band EPR spectrum of RA–Cu(II) complex paired to its best fit simulation; Figure S2: UV–Vis experiments of CA/copper(II) titration in phosphate buffer, Figure S3: Comparison of ^1^H NMR spectra of RA–Cu(II) solutions at different concentrations in absence and in presence of AH.
