# Supplementary material for: A Combined NMR and UV–Vis Approach to Evaluate Radical Scavenging Activity of Rosmarinic Acid and Other Polyphenols
Source: Molecules. 2023 Sep 14;28(18):6629. doi: 10.3390/molecules28186629 (PMC10536562; doi:10.3390/molecules28186629)
Supplement: Supplementary file 1 [file molecules-28-06629-s001.zip › molecules-2572324-supplementary.pdf]

Supplementary Materials

A Combined NMR and UV–Vis Approach to Evaluate Radical Scavenging Activity of Rosmarinic Acid and Other Polyphenols

Arian Kola, Ginevra Vigni, Maria Camilla Baratto and Daniela Valensin *

Department of Biotechnology, Chemistry and Pharmacy, University of Siena, Via Aldo Moro 2, 53100 Siena, Italy; arian.kola@unisi.it (A.K.); ginevra.vigni2@unisi.it (G.V.); mariacamilla.baratto@unisi.it (M.C.B.)

* Correspondence: daniela.valensin@unisi.it; Tel.: +39-0577232428

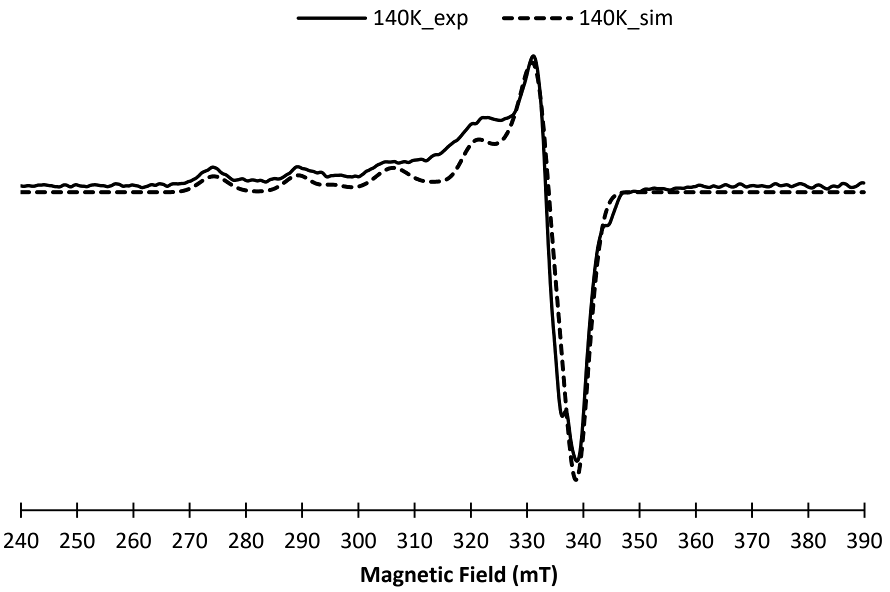

Figure S1. Low temperature (140K) X-band EPR spectrum of RA-Cu(II) complex (black line) paired to its best fit simulation (dotted line). Experimental conditions: ν= 9.67GHz microwave frequency, 0.5mT modulation amplitude, 21mW microwave power.

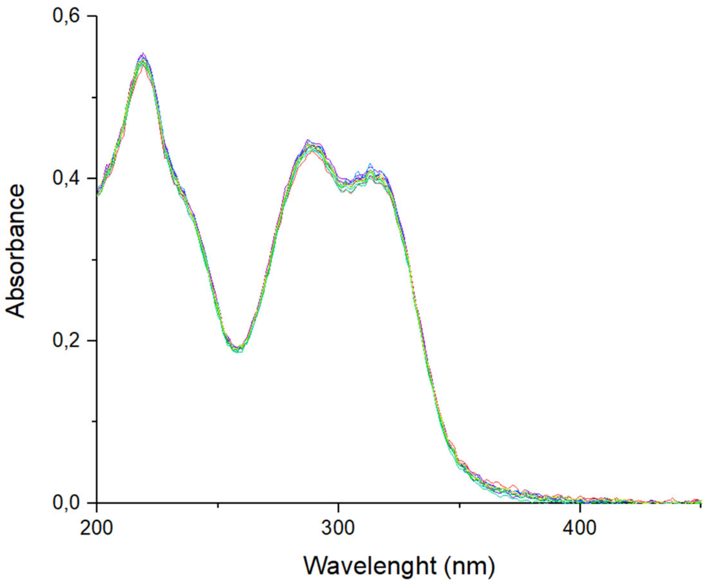

Figure S2. UV-Vis experiments of CA/copper(II) titration in phosphate buffer 1 mM. In orange we can see CA 50 μM in absence of copper(II), while, hereinafter the additions of 0.1 eqs (red), 0.2 eqs (violet), 0.4 eqs (blue), 0.6 eqs (green water), 0.8 eqs (green) and 1 eq (yellow) of copper(II) are reported.

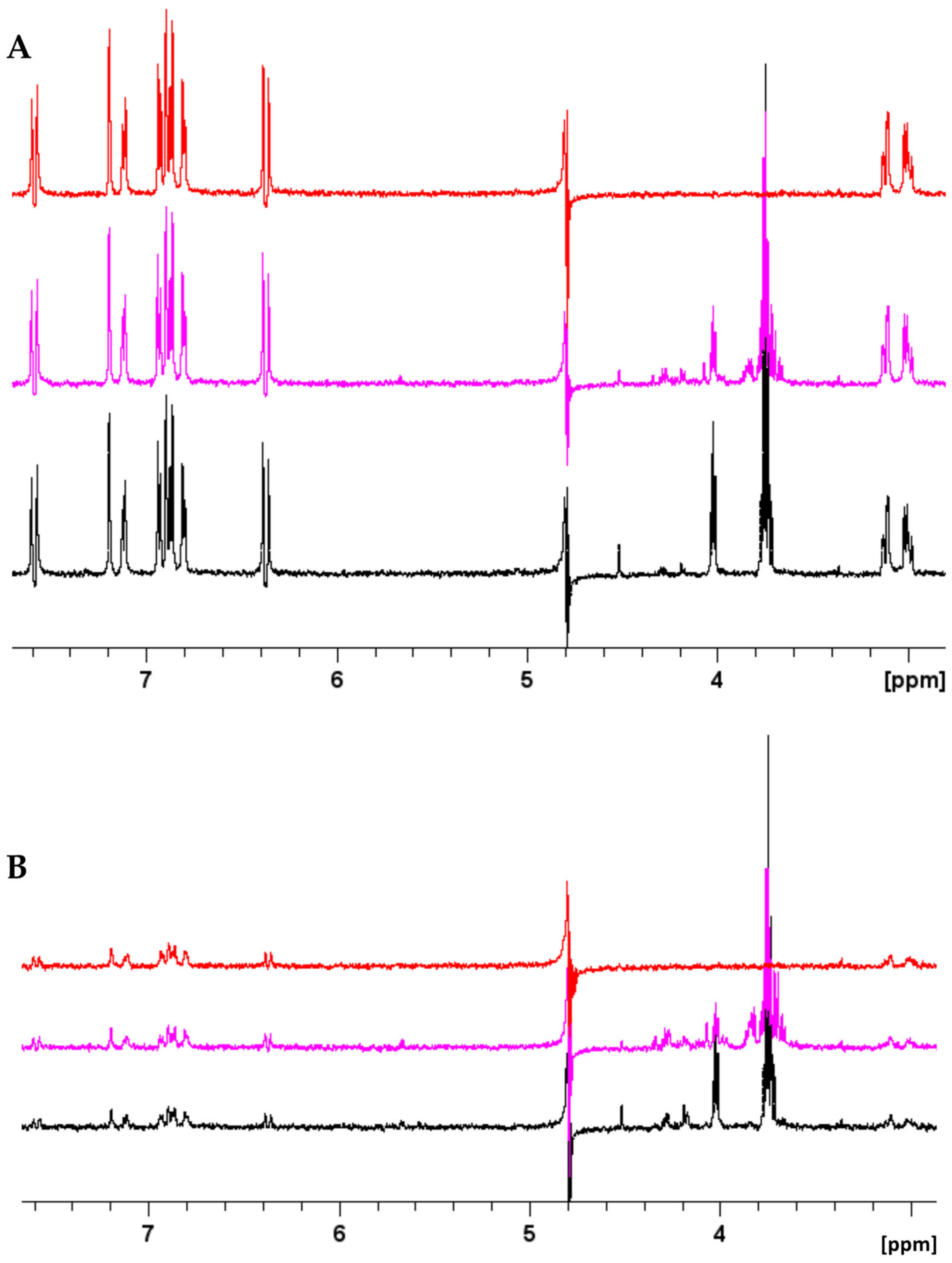

Figure S3. Comparison of ^{1}H NMR spectra of RA–Cu(II) solutions at different concentrations in absence and in presence of AH: A. RA 0.25 mM, Cu(II) 5x10^{-3} mM; B. RA 0.05 mM, Cu(II) 5x10^{-3} mM. In both panels the red spectrum refers to no AH present in solution, the black and magenta ones refers to the presence of 0.5 mM AH at t=0 and t=60′, respectively.
